# Analytical Insights on Theta-Gamma Coupled Neural Oscillators

**DOI:** 10.1186/2190-8567-3-16

**Published:** 2013-08-14

**Authors:** Lorenzo Fontolan, Maciej Krupa, Alexandre Hyafil, Boris Gutkin

**Affiliations:** 1 Department of Fundamental Neurosciences, CMU, University of Geneva, 1 rue Michel Servet, 1211, Geneva, Switzerland; 2 INRIA Paris-Rocquencourt Research Centre, Domaine de Voluceau BP 105, 78153, Le Chesnay, France; 3 Group for Neural Theory, Départment des Etudes Cognitives, Ecole Normale Supérieure, 5 rue d’Ulm, 75005, Paris, France

**Keywords:** Oscillations, PING, Dynamical systems, Geometric singular perturbation theory, Blow-up method, Spike times, Theta-gamma rhythms, Type I neuron, SNIC bifurcation

## Abstract

In this paper, we study the dynamics of a quadratic integrate-and-fire neuron, spiking in the gamma (30–100 Hz) range, coupled to a delta/theta frequency (1–8 Hz) neural oscillator. Using analytical and semianalytical methods, we were able to derive characteristic spiking times for the system in two distinct regimes (depending on parameter values): one regime where the gamma neuron is intrinsically oscillating in the absence of theta input, and a second one in which gamma spiking is directly gated by theta input, i.e., windows of gamma activity alternate with silence periods depending on the underlying theta phase. In the former case, we transform the equations such that the system becomes analogous to the Mathieu differential equation. By solving this equation, we can compute numerically the time to the first gamma spike, and then use singular perturbation theory to find successive spike times. On the other hand, in the excitable condition, we make direct use of singular perturbation theory to obtain an approximation of the time to first gamma spike, and then extend the result to calculate ensuing gamma spikes in a recursive fashion. We thereby give explicit formulas for the onset and offset of gamma spike burst during a theta cycle, and provide an estimation of the total number of spikes per theta cycle both for *excitable* and *oscillator* regimes.

## 1 Introduction

Oscillations of neural activity are ubiquitous in the brain in many frequency bands [1], and it has been often argued that they play a functional role in cortical processing [2-4]. Physiological experiments and computational models have shown that ongoing brain oscillations are involved in sensory-motor functions [5], synaptic plasticity [6], memory formation and maintenance [7], among many other cognitive tasks. Indeed, it has been reported [2] that intrinsic brain rhythms can bias input selection, temporally link neurons into assemblies, and facilitate mechanisms that cooperatively support temporal representation and long-term consolidation of information. Notably gamma oscillations (>30 Hz) are prominent in neocortex during attention [8], sensory processing [9,10], or motor control tasks [11], together with slower rhythms in the theta (3–8 Hz) or delta (1–3 Hz) range that have also been linked to various aspects of cognitive processes like working memory or the transmission of sensory and motor signals. 

Many recent contributions point to nontrivial interactions among different frequency bands [12-14], such as phase-amplitude [15,16] or phase-phase coupling [17,18] that can facilitate the simultaneous integration of multiple layers of information [19]. The hippocampus is a privileged site for observing such interactions [11,20], since theta and gamma waves are particularly strong and reliable in that region [21]. Another particular case is represented by perception of speech signal performed by auditory cortex. In fact, to capture the many different relevant features of speech (i.e., syllables, vowels, consonants, etc.), the brain must be able to parse the speech signal over these many time-scales at the same time. A number of recent works introduced the hypothesis that a network of nested theta (3–8 Hz) and gamma (30–100 Hz) rhythms could accomplish this task [22-24], given their matching in frequency with syllabic and phonemic time-scale, respectively. Since there is no external onset signaling the presence of an incoming syllabic content, the phase of the gamma rhythm needs to be reset by some intrinsic mechanism, e.g., by theta input [23]. It becomes therefore important to know the time to first spike, which would be a measure of the speed of gamma phase resetting, as well as the time to last spike and the spiking frequency during excitable period. 

There is a large literature on mathematical analysis of single frequency oscillators in networks of cortical circuits [25-31], and much work has been done in computational modeling of neural oscillations [2,32,33]. There is also a significant number of mathematical studies on cross-frequency interactions, however, most of that analysis is limited to the cases of weak coupling [34-37]. Strong coupling case has been analyzed either with pulsatile coupling [25,38,39] or with semianalytical and computational techniques [40-42]. Importantly, the question of how strong continuous coupling between slow and fast oscillations influences frequency and time of fast spikes has not been treated analytically, at least to the best of our knowledge. Yet experimental data suggest that phase-amplitude coupling in the brain is continuous (i.e., low-frequency phase is conveyed through local field potential, a continuous signal) and strong [15,39,41], so this will be the regime we aim to study in the present work. 

In this article, we provide analytical insights on the precise spiking times of a simplified Pyramidal Interneuron Network Gamma (PING) [41] during theta modulation. Two separate cases are studied: In the first setting, which we will refer to as *oscillatory regime*, the gamma network behaves as an intrinsic oscillator whose spike frequency is modulated by the theta phase; in the second, named *excitable regime*, gamma spikes are only evoked when input coming from the theta oscillator is strong enough. In the latter case, the system is in an “excitable” regime, where theta pushes gamma back and forth across a Saddle-Node on Invariant Circle (SNIC) bifurcation. The analysis can be generalized beyond theta-gamma nested oscillations; indeed it describes any coupling between low and a high frequency rhythms [43], provided that the latter is produced through feedback inhibition to the excitatory cell. To compute the time to the first gamma spike, we used different approaches for the two regimes: In the oscillatory case, we reduce the system in order to describe its dynamics with the Mathieu equation [44], and in the excitable case we apply an extension of geometric singular perturbation theory [45-47]. We then use a combination of the two to get successive spike times and an estimation of the total number of spikes per theta cycle. 

The paper is organized as follows: 

1. In Sect. 1, we introduce the system to be studied.

2. In Sect. 2, we consider the system in the oscillatory regime and compute time to first gamma spike using Mathieu functions. We found that spike time is mainly determined by the magnitude of theta-gamma coupling (*λ*) and of theta frequency.

3. In Sect. 3, we turn our attention to the excitable regime where theta phase determines the magnitude of input, thereby causing the gamma circuit to spike.

4. Finally, we show that our approach gives results in agreement with direct numerical simulations of the system of interest.

 In our analysis, we use tools from geometric singular perturbation theory. This approach normally fails in proximity of nonhyperbolic points, as it would be the case for the system considered in the present paper, but the *blow-up method* extension provided in [48] allows us to compute approximations of the passage time to the first spike in the excitable case, and it is used both in the oscillator and excitable cases to estimate the duration of inhibition and the passage time of subsequent spikes. The latter estimates are based on the idea that inhibition puts the system in a state of quasi equilibrium; consequently, they work well if inhibition is strong and excitation not too high. 

## 2 Theta-Gamma Coupled Oscillator

We consider a minimal formulation of a theta modulated gamma spiking network. One single Excitatory Gamma (EG) neuron ($\theta_{E}$), modeled as a *θ*-neuron [49] receives an excitatory input coming from an oscillator (*Θ*) whose natural frequency lies in the theta band. The canonical *θ*-neuron model is described by a phase variable lying on a one-dimensional circle in the range $\theta_{E}\in [-\pi ,\pi )$, a spike is produced when $\theta_{E}=\pi$. The EG neuron participates in a PING rhythm, although in our case the inhibitory gamma neuron is instantaneously enslaved to the excitatory cell, meaning that every excitatory spike would immediately prompt a simultaneous inhibitory spike [32]. This allows us to suppress the explicit dynamics of the inhibitory gamma neuron and focus on inhibitory synaptic dynamics only. Our system can be described by the following equations:

(1) $$\begin{array}{rl} \frac{d\theta_{E}}{dt} & =\left(1-\cos(\theta_{E})\right)+\left(I_{E}+\lambda \left(1+\cos(\Theta )\right)-g_{EI}s_{I}\right)\left(1+\cos(\theta_{E})\right),\\ \frac{ds_{I}}{dt} & =-\varepsilon_{I}s_{I}+\delta (\theta_{E}-\pi ),\\ \frac{d\Theta }{dt} & =\varepsilon_{\Theta }\omega , \end{array}$$

where Θ∈[−π,π) is the instantaneous phase of the slow rhythm variable (delta/theta frequency band, i.e., 1–8 Hz), which provides the sinusoidal modulatory input to the EG cell; $s_{I}$ is the variable representing the activation of the inhibitory synapse; $I_{E}$ represents constant driving input to excitatory gamma neuron; *λ* is the strength of theta-gamma coupling; $g_{EI}$ is the inhibitory synaptic strength; *ω* has been chosen so that frequency $\varepsilon_{\Theta }\omega$ falls into the theta range; $\varepsilon_{I}$ is a scaling parameter that scales inversely with the time constant of synaptic inhibition; $\varepsilon_{\Theta }$ is a second, slower, scaling parameter that has been chosen such that $\varepsilon_{\Theta }∼\varepsilon_{I}^{2}$, an assumption that is motivated by biophysical considerations and, in addition, keeps the three time scales (theta rhythm, synaptic inhibition, and excitatory membrane potential) well separate.

We will consider two cases: the *oscillator* case, defined by $I_{E}>0$, and the *excitable* case, defined by $I_{E}<0$. The characterizing feature of the oscillator setting is that $\theta_{E}$–$s_{I}$ subsystem in (1) is an intrinsic oscillator at every stage of a *Θ*-cycle, i.e., the total current input to EG neuron is always positive. In the *excitable* case, on the other hand, part of theta oscillation period is such that $\theta_{E}$ subsystem of (1) has an attracting quasisteady state, i.e., the total input to the EG neuron is negative or positive depending on *Θ*-oscillator phase. If $I_{E}<-2\lambda$, the net input to EG neuron is always negative and the gamma circuit is always silent.

## 3 Time to First Spike, Oscillator Case

Let us consider the case in which constant driving term $I_{E}$ in system (1) is positive and such that, in absence of theta modulation, the EG neuron would fire periodically with a spiking frequency in the gamma range (30–150 Hz). We assume that the dynamics of the theta oscillator is at least one order of magnitude slower than synaptic decay, so that almost no residual inhibition is present at the beginning of a new theta cycle. In order to obtain an equation in the form of Mathieu equation, we first perform a change of variables in system (1) $V_{E}=\tan\frac{\theta_{E}}{2}$, going from *θ*-variable to membrane voltage $V_{E}$. As it is known [40], *θ*-neuron is formally equivalent to the Quadratic Integrate and Fire (QIF) neuron:

(2) $$\begin{array}{rl} \frac{dV_{E}(t)}{dt} & =V_{E}^{2}(t)+I_{E}+\lambda \left(1+\cos(\Theta )\right),\\ \frac{d\Theta }{dt} & =\varepsilon_{\Theta }\omega . \end{array}$$

The two neural models are formally equivalent if we define the reset conditions as

$$V_{E}\left(t^{*}-0\right)=+\infty ,\;V_{E}\left(t^{*}+0\right)=-\infty ,$$

where $t^{*}$ the time of spike. We have omitted the synaptic input dynamics since we assume that inhibition is directly enslaved to spikes coming from EG neuron, hence the inhibitory synapse $s_{I}$ stays inactive up to the first EG spike. We restate the system in (2) as a single equation, assuming by convention that Θ=−π at t=0 (or at the beginning of a new theta cycle):

(3) $$\frac{dV_{E}(t)}{dt}=V_{E}^{2}(t)+I_{E}+\lambda \left(1+\cos(\varepsilon_{\Theta }\omega t-\pi )\right).$$

For $I_{E}>0$, this equation has an exact solution in terms of Mathieu functions. This can be found by imposing one more change of variable:

(4) $$V_{E}=-\frac{u^{\prime }}{u},\;u(t)=e^{-\int V_{E}(\tau )d\tau },$$

where the prime mark denotes the time derivative (a similar transformation is used in [50] where the cosinusoidal forcing term was replaced by exponential decay, leading to a different solution of the corresponding differential equation). Hence, we write (3) as a second-order differential equation:

(5) $$u^{\prime \prime }=-\left(I_{E}+\lambda \left(1+\cos(\varepsilon_{\Theta }\omega t-\pi )\right)\right)u.$$

If parameters *a*, *q*, *z* are rescaled as following:

(6) $$z=\frac{\varepsilon_{\Theta }\omega t}{2},\;a=\frac{4(I_{E}+\lambda )}{\varepsilon_{\Theta }^{2}\omega^{2}},\;q=\frac{2\lambda }{\varepsilon_{\Theta }^{2}\omega^{2}},$$

then Eq. (5) has the form of a Mathieu equation:

(7) $$\frac{d^{2}u}{dz^{2}}=-\left(a-2q\cos(2z)\right)u.$$

**Fig. 1:**
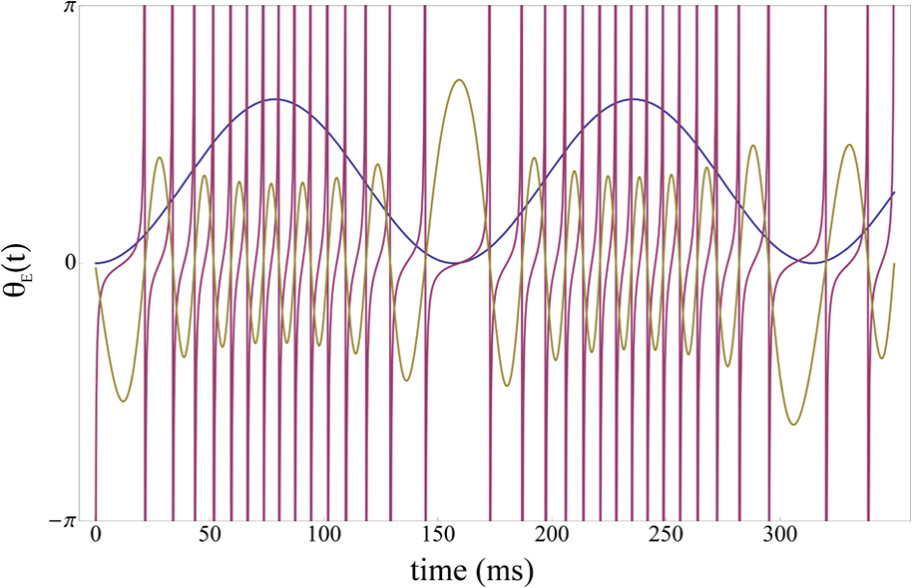
Oscillatory regime—dynamics of theta-modulated EG neuron in absence of inhibition. *Red line*: membrane voltage of EG neuron in presence of theta modulation (*blue line*) without inhibitory synaptic input ($g_{IE}=0$). Solution u(t) to Mathieu equation is plotted in *yellow*

**Fig. 2:**
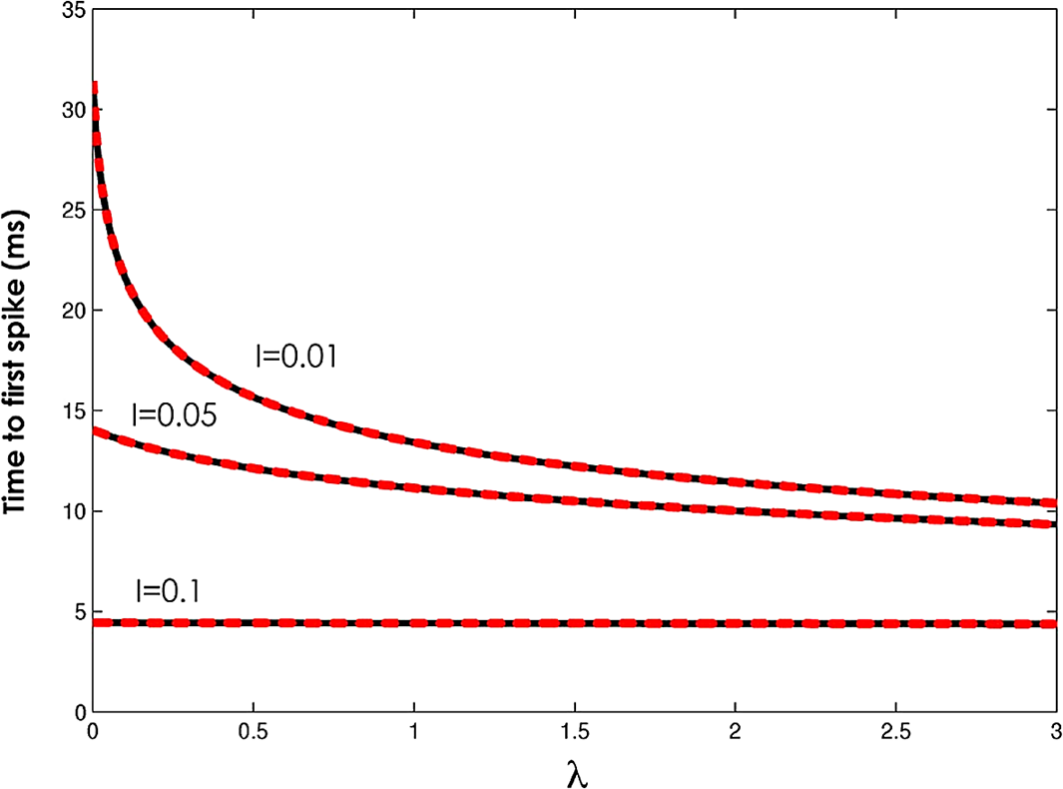
Oscillatory regime—time to first spike. Time to first spike as a function of coupling constant *λ*, for $I_{E}=0.01$, $I_{E}=0.05$ and $I_{E}=0.1$. *Black line*: simulation; *red dotted line*: solution to Mathieu equation. $\varepsilon_{\Theta }=0.01$, ω=4

To interpret Eq. (7), we need temporal rescaling from *t* to *z*, and as a consequence the period of cosinusoidal term, which in Eqs. (1) and (2) was $T=\frac{2\pi }{\varepsilon_{\Theta }\omega }$, becomes $T^{*}=\pi$. The solutions to Eq. (7) are linear combinations of the even and odd Mathieu functions [44], Ce(a,q,z) and Se(a,q,z), respectively. The solution

$$\begin{array}{rl} u(z)= & 2(-2\pi \mathrm{Ce}(a,q,0)+\varepsilon_{\Theta }\omega \dot{\mathrm{Ce}}(a,q,0)\mathrm{Se}(a,q,z)\\ & +\mathrm{Ce}(a,q,z)\left(4\pi \mathrm{Se}(a,q,0)-2\varepsilon_{\Theta }\omega \dot{\mathrm{Se}}(a,q,0)\right)), \end{array}$$

obeys the desired initial conditions, where the dot indicates the derivative with respect to *z*. Because of the change of variable in (4), the spiking times in the absence of inhibition correspond to the zeros of the solution of (7) u(z) (Fig. 1). Hence, by scaling back to the original variables and looking at the first zero of u(z) we obtain the time to the very first spike $T_{1}$. We numerically compute the time to the first spike as a function of parameters *a* and *q*, i.e., $I_{E}$ and *λ*. The subsequent spikes, on the other hand, depend on inhibition, and thus cannot be described by (3) alone. We looked for solutions of (2) with initial condition $V_{E}(0)=-\infty$ and Θ(0)=−π. Figure 2 shows the time to first spike $T_{1}$ as a function of *λ* with $I_{E}$ fixed at three values, $I_{E}=0.01$, $I_{E}=0.05$, and $I_{E}=0.1$. Note that for $I_{E}=0.01$ the dependence on *λ* is strong, but for larger values of $I_{E}$ the sensitivity of $T_{1}$ with respect to *λ* is smaller, since $I_{E}$ becomes the dominant input term. In the next section, we will consider the case when inhibition is strong and fully controls the gamma spikes. 

## 4 Time to First Spike, Excitable Case

The excitable case implies that $I_{E}<0$, and $2\lambda +I_{E}>0$. Under these assumptions, the gamma spikes are only possible when *Θ* lies in a proper subinterval of [−π,π), which corresponds to the values of *Θ* for which $\lambda (1+\cos(\Theta ))+I_{E}>0$. This ensures that the dynamics of (1) cross the SNIC bifurcation for a certain value of cos(Θ). We carry out the computation with the initial conditions

(8) $$\theta_{E}=\theta_{0},\;\Theta =-\pi ,\;s_{I}=0,$$

where $-\pi <\theta_{0}<0$ is defined by the condition $(1-I_{E})\cos\theta_{0}=1+I_{E}$, i.e. $\theta_{0}$ is a stationary fixed point in the absence of *Θ* positive input. The gamma neuron relaxes to $\theta_{0}$ when theta modulation is turned off. Note that $\theta_{E}=\theta_{0}$ is not required for our solution to be applicable, since, for any initial value $\theta_{\mathrm{in}}<\theta_{0}$, $\theta_{E}$ quickly converges to $\theta_{0}$. At the end of every theta cycle $s_{I}$ goes back to zero, since its decay constant $\varepsilon_{I}$ is one order of magnitude bigger than $\varepsilon_{\Theta }$, and the EG cell has stopped firing once inhibition pushed it below the SNIC bifurcation. We start by computing an estimate of the time to the first gamma spike. System (1) involves two time scales, one that controls the intrinsic dynamics of the EG neuron and the other comes from *Θ* modulation. In the excitable case, rather than using the approach based on Mathieu functions, we use geometric singular perturbation theory. This approach leads to explicit estimates of the onset and duration of the gamma burst, it gives some geometric insights and can be applied in a more general setting. In order to compute the time at which the fast and the slow dynamics intersect, we need the value of *Θ* corresponding to $I_{E}+\lambda (1+\cos(\Theta ))=0$, i.e., where the SNIC bifurcation takes place. Simple algebra shows that this occurs when

(9) $$\cos(\Theta )=-\frac{\lambda +I_{E}}{\lambda }.$$

To ensure that (9) has solutions, we verify that the RHS of (9) is in the interval (−1,1). For the upper bound, we have

$$2\lambda +I_{E}>0\;\Rightarrow \;\lambda >-(I_{E}+\lambda )\;\Rightarrow \;1>-\frac{\lambda +I_{E}}{\lambda }.$$

**Fig. 3:**
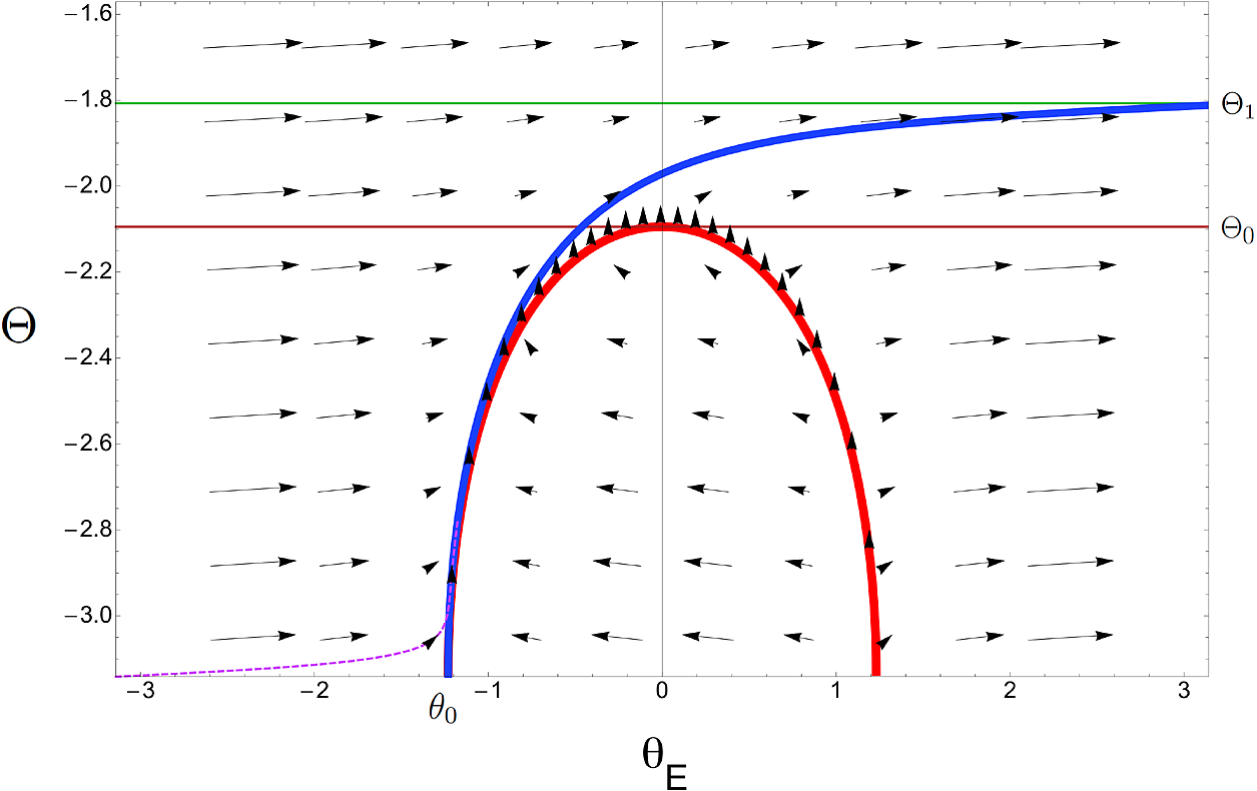
Excitatory regime—phase plane. Phase portrait of system (1) for $I_{E}=-0.5$, λ=1, $\varepsilon_{\Theta }=0.01$, ω=4. *The blue line* represents the trajectory of the system when starting from initial conditions $\left(\theta_{0},-\pi \right)$: it passes along the nullcline $\frac{d\theta_{E}}{dt}=\varphi (\theta_{E},\Theta )=0$ (in *red*) and then quickly escapes to $\left(\pi ,\Theta_{1}\right)$ once past the singular point $\left(0,\Theta_{0}\right)$. *The dotted purple line* shows that, for any starting point $\left(\Theta ,\theta_{E}\right)$ where $\Theta <\Theta_{0}$ and $\theta_{E}<\theta_{0}$, the trajectory converges to *the blue line*

The lower bound is obtained as follows:

$$-\frac{\lambda +I_{E}}{\lambda }=-1+\frac{-I_{E}}{\lambda }>-1$$

given that $\frac{-I_{E}}{\lambda }>0$. Let now $\Theta_{0}$ be a solution of (9) satisfying $-\pi <\Theta_{0}<0$ and let us consider system (1) with initial conditions (8): it is clear that any trajectory of the system can roughly be divided into two separate chunks (see Fig. 3). Starting from point $\left(\theta_{0},-\pi \right)$, the system immediately enters the slow motion part of the trajectory, which is adjacent to the nullcline $\frac{d\theta_{E}}{dt}=\varphi (\theta_{E},\Theta )=0$. The slowest region of motion lies in the vicinity of the singular point $\left(0,\Theta_{0}\right)$, where both $\varphi (\theta_{E},\Theta )$ and its derivative with respect to $\theta_{E}$ are zero. Once the trajectory has gone beyond the singular point, *φ* turns positive again and grows quadratically in magnitude. This way $\theta_{E}$ quickly reaches the value $\theta_{E}=\pi$, since it is well known that any unbounded solution of the theta neuron for positive net input explodes in finite time. At the same time *Θ* increase is of order $∽O(\varepsilon_{\Theta })$. Now let us start by computing the time spent along the fiber which is close to the nullcline, and then direct our attention to the motion in the neighborhood of the singular point. The time needed for *Θ* to grow from −*π* to $\Theta_{0}$ equals

(10) $$T_{1}^{*}=\frac{\Theta_{0}+\pi }{\varepsilon_{\Theta }\omega }.$$

When *Θ* reaches $\Theta_{0}$, $\theta_{E}$ is $O(\varepsilon_{\Theta })$ (recall that this is also $O(\varepsilon^{2})$) close to the threshold value of $\theta_{E}=0$. In order to estimate the time that EG neuron needs to produce the first spike, i.e., to reach $\theta_{E}=\pi$, we need to examine the behavior when close to point $\left(0,\Theta_{0}\right)$. We first translate the variable *Θ*, introducing $\tilde{\Theta }=\Theta -\Theta_{0}$. Using Taylor expansion around $\theta_{E}\approx 0$ and $\Theta \approx \Theta_{0}$, we can write

(11) $$\begin{array}{rl} 1-\cos(\theta_{E}) & ≃\frac{1}{2}\theta_{E}^{2}+O\left(\theta_{E}^{4}\right),\\ I_{E}+\lambda \left(1+\cos(\Theta )\right) & ≃\sqrt{-I_{E}(2\lambda +I_{E})}\tilde{\Theta }+O\left({\tilde{\Theta }}^{2}\right). \end{array}$$

We transform (1) to the coordinates $\left(\theta_{E},\tilde{\Theta }\right)$, taking into the account the expansion (11) and ignoring $s_{I}$ which remains zero until the first gamma spike (we omit the tilde for the simplicity of notation). The resulting system is

(12) $$\begin{array}{rl} \frac{d\theta_{E}}{dt} & =a\Theta +\frac{1}{2}\theta_{E}^{2}+O\left(\theta_{E}^{4},\Theta^{2},\theta_{E}^{2}\Theta \right),\\ \frac{d\Theta }{dt} & =\varepsilon_{\Theta }\omega , \end{array}$$

with $a=2\sqrt{-I_{E}(2\lambda +I_{E})}$. 

We then rescale the variables of (12) as follows:

$$\theta_{E}=2x,\;\Theta =\frac{2}{a}y,\;\tilde{\varepsilon }=\frac{\varepsilon_{\Theta }\omega a}{2}.$$

In terms of the rescaled variables, with the tilde omitted, system (12) becomes

(13) $$\begin{array}{rl} \frac{dx}{dt} & =f(x,y)=y+x^{2}+O\left(x^{4},y^{2},x^{2}y\right),\\ \frac{dy}{dt} & =\varepsilon . \end{array}$$

This means that the nullcline of system (13) for ε=0, defined by the parabola f(x,y)=0, is a good approximation for the nullcline of system (1) in the neighborhood of the singular point $\left(0,\Theta_{0}\right)$, or equivalently, in coordinates (x,y), the point (0,0). Thus system (13) has the same form as system (2.5) in [48] and can be restated as a Riccati equation:

(14) $$\frac{dx}{dy}=\frac{y+x^{2}}{\varepsilon }.$$

By performing a change of variable, it can be shown that (14) is equivalent to a second-order Bessel equation. The following function is a general solution of (14):

(15) $$x=\zeta (y)=-\sqrt{v}\frac{J_{-2/3}(2y^{3/2}/3)-cJ_{2/3}(2y^{3/2}/3)}{cJ_{-1/3}(2y^{3/2}/3)+J_{1/3}(2y^{3/2}/3)},$$

where $J_{\nu }$ are Bessel functions of the first kind of order *ν*. The only solution approaching the left branch of the nullcline parabola for y<0 is the one obtained by choosing c=1, thus we pick this value of *c*. The inverse of function ζ(y), namely $\xi (x)=\zeta^{-1}(y)$, defines the trajectory of *x* as a function of *y* (Fig. 3). Unfortunately, due to its highly nonlinear form, it is impossible to compute directly.

We use these results together with Theorem 2.1 and Remark 2.11 in [48] in order to derive the following estimate (the result dates back to much earlier, see for example [51]). 

**Proposition 1***Let*$y_{0}>0$*and*$x_{0}<0$*satisfy*$f(x_{0},y_{0})=0$. *Also fix*δ>0. *Consider a family of solutions of* (13) *with initial conditions*$x(0)=x_{0}+O(\varepsilon )$*and*$y(0)=y_{0}$. *Let*(δ,h(ε))*be the intersection point of this trajectory with the line*x=δ. *Then*, *for sufficiently smallδ*,

(16) $$h(\varepsilon )=\Omega_{0}\varepsilon^{2/3}+O(\varepsilon \ln\varepsilon ),$$

*where*$\Omega_{0}$*stands for the smallest positive zero of the denominator in* (15):

$$J_{-1/3}\left(\frac{2y^{3/2}}{3}\right)+J_{1/3}\left(\frac{2y^{3/2}}{3}\right).$$

From now on, we will use the numerical approximation

$$\Omega_{0}\approx -2.34.$$

Note that the solution with initial conditions (8), transformed to the coordinates (x,y), satisfies the assumptions of Proposition 1. Therefore, estimate (17) holds.

Now let $T_{1}$ be the time the of the first gamma spike, i.e., when $\theta_{E}=\pi$. From (16), it is easy to see that, after scaling back to the original variables ($\theta_{E}$, *Θ*, $\varepsilon_{\Theta }$), $T_{1}$ can be written as

(17) $$T_{1}=\frac{\Theta_{0}+\pi }{\varepsilon_{\Theta }\omega }+\frac{C_{0}}{\varepsilon_{\Theta }^{1/3}}+O(\ln\varepsilon_{\Theta }),$$

with

(18) $$C_{0}=-\frac{2^{1/3}\Omega_{0}}{{\left(\omega a\right)}^{1/3}}.$$

The $O(\ln\varepsilon_{\Theta })$ term in (17) and the following one, of order O(1) in $\varepsilon_{\Theta }$, happen to be zero in the theta neuron model (as well as in the QIF model) when there is no excitatory feedback from the EG cell to the theta band oscillator (see the Appendix). The next nonzero term in (17) is then of order $O(\varepsilon_{\Theta }^{1/3})$, which represents the error with respect to the time at which the true trajectory of the system reaches $\theta_{E}=2\delta$. The value of *δ* does not have to be small, on the contrary our approximation works better when *δ* is such that the trajectory of the system is close to the asymptote $\Theta =C_{0}\omega \varepsilon_{\Theta }^{2/3}$, as it is the case for the EG cell spike threshold $\delta =\frac{\pi }{2}$.

**Fig. 4:**
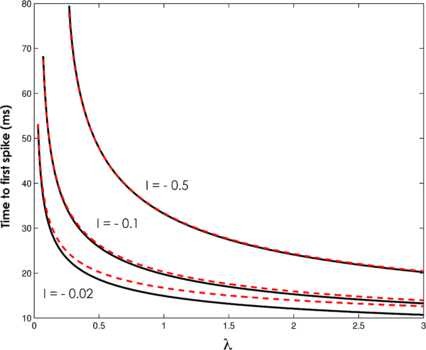
Excitable regime—time to first spike. Time to first spike as a function of coupling constant *λ*, for $I_{E}=-0.5$, $I_{E}=-0.1$ and $I_{E}=-0.02$. *Black full line*: simulation; *red dotted line*: analytic solution. $\varepsilon_{\Theta }=0.01$, ω=4

Predictions of Proposition 1 are illustrated in Figs. 3 and 4. 

## 5 Subsequent Gamma Spikes, Oscillator Case

In the oscillator case, we assume that inhibition is strong enough to push the system below the SNIC bifurcation, regardless of the value of *Θ*, i.e.,

(19) $$g_{IE}>I_{E}+2\lambda .$$

If the opposite is true, the system does not encounter the bifurcation, since $\frac{d\theta_{E}}{dt}$ is always greater than zero, and our analysis cannot be applied to subsequent spikes. We wish to derive an estimate on the number of EG spikes occurring along one *Θ* period, which is given by the time needed for *Θ* to grow from −*π* to *π*, thus equal to $2\pi /(\varepsilon_{\Theta }\omega )$. Let $T_{2},\ldots ,T_{L}$ be the subsequent gamma spikes and $\Theta_{2},\ldots ,\Theta_{L}$, the corresponding values of *Θ*. Let $T_{j}^{*}$ be the relative time after $T_{j-1}$ at which the total driving input to the EG neuron reaches zero from negative values:

(20) $$I_{E}+\lambda \left(1+\cos\left(\Theta_{j-1}+\varepsilon_{\Theta }\omega T_{j}^{*}\right)\right)-g_{IE}e^{-\varepsilon_{I}T_{j}^{*}}=0.$$

From now on, we use the fact that $\varepsilon_{\Theta }\approx \varepsilon_{I}^{2}$, and relabel $\varepsilon_{I}\equiv \varepsilon$. Hence, we can write

(21) $$\cos\left(\Theta_{j-1}+\varepsilon^{2}\omega T_{j}^{*}\right)\approx \cos(\Theta_{j-1})-\sin(\Theta_{j-1})\varepsilon^{2}\omega T_{j}^{*}.$$

We expect $T_{j}^{*}$ to be of order $O(\varepsilon^{-1})$ from (20), $\cos(\Theta_{j-1})$ is then large compared to $\sin(\Theta_{j-1})\varepsilon^{2}\omega T_{j}^{*}$. We can thus replace (20) by a simpler formula:

(22) $$e^{\varepsilon T_{j}^{*}}=\frac{g_{IE}}{I_{E}+\lambda (1+\cos(\Theta_{j-1}))}.$$

Now

(23) $$T_{j}^{*}=\frac{1}{\varepsilon }\ln\left(\frac{g_{IE}}{I_{E}+\lambda (1+\cos(\Theta_{j-1}))}\right).$$

We denote the time interval between two successive gamma spikes by $\Delta T_{j}$ and use the following estimate:

$$\Delta T_{j}=T_{j}-T_{j-1}\approx T_{j}^{*}+T_{j}^{**},$$

where

(24) $$T_{j}^{**}=C_{j}\varepsilon^{-1/3},\;C_{j}=-\frac{\Omega_{0}}{{\left(I_{E}+\lambda (1+\cos\Theta_{j-1})\right)}^{1/3}}.$$

Estimate (24) is obtained analogously as (17). We can write the modulated instantaneous Interspike Interval (ISI), i.e., the instantaneous period, as

(25) $$\Delta T_{j}=\frac{1}{\varepsilon }\ln\left(\frac{g_{IE}}{I_{E}+\lambda (1+\cos(\Theta_{j-1}))}\right)-\frac{1}{\varepsilon^{1/3}}\frac{\Omega_{0}}{{\left(I_{E}+\lambda (1+\cos\Theta_{j-1})\right)}^{1/3}}.$$

We derive the intrinsic period of PING in absence of any modulation:

(26) $$\Delta T_{j}^{IN}=\frac{1}{\varepsilon }\ln\left(\frac{g_{IE}}{I_{E}}\right)-\frac{\Omega_{0}}{\varepsilon^{1/3}I_{E}}.$$

After some algebra and performing a second-order Taylor expansion around $\Theta_{j-1}\approx -\pi$, i.e., when theta excitation is minimal, the ISI becomes

(27) $$\Delta T_{j}^{\max}=\Delta T_{j}^{IN}-\frac{1}{\varepsilon }\frac{\lambda }{2I_{E}}{\left(\Theta_{j-1}+\pi \right)}^{2}-\frac{1}{\varepsilon^{1/3}}\left[\frac{\Omega_{0}\lambda }{6I_{E}^{4/3}}{\left(\Theta_{j-1}+\pi \right)}^{2}\right].$$

The smallest ISI is obtained by expanding around $\Theta_{j-1}\approx 0$, i.e., when theta excitation is maximal:

(28) $$\begin{array}{rl} \Delta T_{j}^{\min}= & \Delta T_{j}^{IN}-\frac{1}{\varepsilon }\left[\ln\left(\frac{I_{E}+2\lambda }{I_{E}}\right)-\frac{\lambda }{2(I_{E}+2\lambda )}\Theta_{j-1}^{2}\right]\\ & -\frac{1}{\varepsilon^{1/3}}\left[\Omega_{0}\left(\frac{1}{{\left(I_{E}+2\lambda \right)}^{1/3}}-\frac{1}{I_{E}^{1/3}}+\frac{\lambda }{6{\left(I_{E}+2\lambda \right)}^{4/3}}\Theta_{j-1}^{2}\right)\right], \end{array}$$

from (27) and (28) we can estimate respectively the lowest and highest gamma frequencies attained during theta modulation. We then derive an expression for the interval of time Δ*T* between the first to the last gamma spike within half a *Θ*-period:

(29) $$\begin{array}{rl} \Delta T= & \frac{1}{\varepsilon }\sum_{i=1}^{M_{0}}\ln\left(\frac{g_{IE}}{I_{E}+\lambda (1+\cos(\Theta_{j-1}))}\right)\\ & +\frac{1}{\varepsilon^{1/3}}\sum_{i=1}^{M_{0}}\frac{-\Omega_{0}}{{\left(I_{E}+\lambda (1+\cos(\Theta_{j-1}))\right)}^{1/3}}, \end{array}$$

where $M_{0}$ is the number of gamma spikes as *Θ* varies between −*π* and 0. Writing

$$\frac{1}{M_{0}}\sum_{i=1}^{M_{0}}\ln\left(\frac{g_{IE}}{I_{E}+\lambda (1+\cos(\Theta_{j-1}))}\right)\approx \frac{1}{\pi }\int_{-\pi }^{0}\ln\left(\frac{g_{IE}}{I_{E}+\lambda (1+\cos(\Theta ))}\right)d\Theta$$

and

$$\frac{1}{M_{0}}\sum_{i=1}^{M_{0}}\frac{-\Omega_{0}}{{\left(I_{E}+\lambda (1+\cos(\Theta_{j-1}))\right)}^{1/3}}\approx \frac{1}{\pi }\int_{-\pi }^{0}\frac{-\Omega_{0}}{{\left(I_{E}+\lambda (1+\cos(\Theta ))\right)}^{1/3}}d\Theta$$

we obtain

(30) $$\begin{array}{rl} & \frac{M_{0}}{\pi }(\varepsilon \int_{-\pi }^{0}\ln\left(\frac{g_{IE}}{I_{E}+\lambda (1+\cos(\Theta ))}\right)d\Theta \\ & \;+\varepsilon^{5/3}\int_{-\pi }^{0}\frac{-\Omega_{0}}{{\left(I_{E}+\lambda (1+\cos(\Theta ))\right)}^{1/3}}d\Theta )\approx \frac{\pi }{\omega }. \end{array}$$

As $M_{0}$ provides an estimate for the number of gamma spikes as *Θ* grows from −*π* to 0, the total number of gamma spikes *M* is the largest integer such that

(31) $$\begin{array}{rl} & \frac{M}{2\pi }(\varepsilon \int_{-\pi }^{\pi }\ln\left(\frac{g_{IE}}{I_{E}+\lambda (1+\cos(\Theta ))}\right)d\Theta \\ & \;+\varepsilon^{5/3}\int_{-\pi }^{\pi }\frac{-\Omega_{0}}{{\left(I_{E}+\lambda (1+\cos(\Theta ))\right)}^{1/3}}d\Theta )<\frac{2\pi }{\omega }. \end{array}$$

**Fig. 5:**
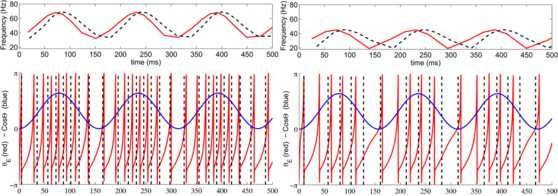
Oscillatory regime—dynamics. Two cases in which formula (31) gives a correct prediction of the number of gamma spikes and a fair estimate of spike times. *Upper panel*: instantaneous firing frequency of gamma cell obtained from simulation (*full red line*) and from Eq. (25) (*black dotted line*). *Lower panel*: the simulation of EG cell membrane potential is shown in *red* while *black dotted lines* represent firing times predicted by our analysis; *the blue curve* shows theta modulation (1+cosΘ). *Left*: $I_{E}=0.5$, ε=0.1, λ=0.8, $g_{IE}=6$, ω=4. *Right*: $I_{E}=0.1$, ε=0.1, λ=0.5, $g_{IE}=6$, ω=4

**Fig. 6:**
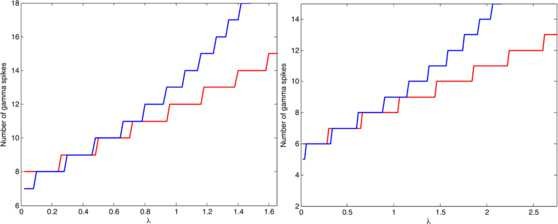
Oscillatory regime—number of spikes. Number of spikes in the simulation (*blue*) and the prediction of the formula as a function of *λ* varying from 0 to $(g_{IE}-I_{E})/2$, ω=4. *In the left panel*$I_{E}=0.7$, ε=0.1, $g_{IE}=4$. *In the right panel*$I_{E}=0.5$, ε=0.1, $g_{IE}=6$

This formula works well, especially when inhibition is sufficiently strong. Figure 5 shows two cases where the formula gives the exact prediction of the number of gamma spikes and a good approximation of spike times. It is worth to mention that in the oscillatory case there is no phase reset at the end of a theta cycle, meaning that the initial conditions are never the same at the beginning of a theta oscillation. As a consequence, the result in (31) does not hold as a rigorous solution but as an average estimate, and the exact number of spikes can still vary over different trials. In Fig. 6, we show the direct comparison between the predictions of the formula and the simulation, as a function of *λ*. Note that for *λ* very small the estimate of the formula is too big. There we would need to include more terms in the *ε* expansion to get a more accurate prediction. When *λ* is large, for fixed $g_{IE}$, the positive input is such that inhibition is not sufficient to periodically time the spikes. As a consequence, the estimate of formula (31) becomes too small. 

## 6 Subsequent Gamma Spikes, Excitable Case

### 6.1 Second Gamma Spike

Let $T_{2}^{*}$, be defined by

(32) $$I_{E}+\lambda \left(1+\cos\left(\Theta \left(T_{1}+T_{2}^{*}\right)\right)\right)-g_{IE}e^{-\varepsilon T_{2}^{*}}=0.$$

Note that

(33) $$\Theta \left(T_{1}+T_{2}^{*}\right)=\Theta_{1}+\omega \varepsilon^{2}T_{2}^{*}=\Theta_{0}+\omega \varepsilon^{2}T_{2}^{*}+O\left(\varepsilon^{4/3}\right)+O\left(\varepsilon^{2}\ln\varepsilon^{2}\right).$$

but, similarly to the oscillator case, we expect $T_{2}^{*}$ to be of order $O(\varepsilon^{-1})$ from (32). This allows us to neglect the last two terms on the RHS of (33). Hence, by (32) and (33), we have

(34) $$e^{\varepsilon T_{2}^{*}}=\frac{g_{IE}}{\lambda (-\sin(\Theta_{0})\varepsilon^{2}\omega T_{2}^{*}+O(\varepsilon^{4/3}))}.$$

Further, we write

(35) $$\varepsilon T_{2}^{*}=-\ln\varepsilon -\ln(-\ln\varepsilon )+A,$$

and substitute into (34) getting

(36) $$\begin{array}{rcl} -\frac{1}{\varepsilon \ln\varepsilon }e^{A} & = & \frac{g_{IE}}{-\lambda \sin(\Theta_{0})\omega \varepsilon (-\ln\varepsilon -\ln(-\ln(\varepsilon ))+A)}\\ & = & \left(-\frac{1}{\varepsilon \ln\varepsilon }\right)\left(\frac{g_{IE}}{-\lambda \sin(\Theta_{0})\omega }\right)\frac{1}{\left(1-(-\ln(-\ln(\varepsilon ))+A)/\ln\varepsilon \right)}\\ & \approx & \left(-\frac{1}{\varepsilon \ln\varepsilon }\right)\left(\frac{g_{IE}}{-\lambda \sin(\Theta_{0})\omega }\right)\left(1-\frac{\ln(-\ln(\varepsilon ))}{\ln\varepsilon }+\frac{A}{\ln\varepsilon }\right); \end{array}$$

(37) $$e^{A}\approx -\frac{g_{IE}}{\lambda \sin(\Theta_{0})\omega }+O\left(-\frac{\ln(-\ln(\varepsilon ))}{\ln\varepsilon }\right).$$

It then follows from (35) that keeping only the leading terms:

(38) $$T_{2}\approx -\frac{\ln\varepsilon }{\varepsilon }+T_{1}\;\text{and}\;\Theta_{2}\approx -\omega \varepsilon \ln\varepsilon +\Theta_{1}.$$

### 6.2 Subsequent Gamma Spikes

We can write a variant of estimate (31) for the excitable case:

(39) $$\begin{array}{rl} & \frac{M}{2\Theta_{0}}(\varepsilon \int_{\Theta_{0}}^{2\pi -\Theta_{0}}\ln\left(\frac{g_{IE}}{I_{E}+\lambda (1+\cos(\Theta ))}\right)d\Theta \\ & \;+\varepsilon^{5/3}\int_{\Theta_{0}}^{2\pi -\Theta_{0}}\frac{1}{{\left(I_{E}+\lambda (1+\cos(\Theta ))\right)}^{1/3}}d\Theta )<\frac{2\Theta_{0}}{\omega }, \end{array}$$

where now the extremes in the integrals are chosen to be the times of the first and last gamma spikes (i.e., the times when the EG neuron crosses the SNIC bifurcation respectively from below and above), assuming that these would be approximately symmetric with respect to the *Θ* cycle.

**Fig. 7:**
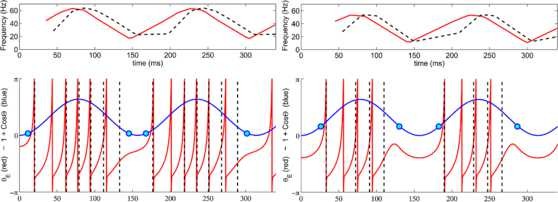
Excitable regime—dynamics. Three cases for which formula (31) gives a correct prediction of the number of gamma spikes. *Plot colors* as in Fig. 4. $\Theta_{0}$ and $2\pi -\Theta_{0}$, i.e. the theta phases where the first and last Hopf bifurcation approximately take place, are shown in *cyan*. ω=4, ε=0.1 and $g_{IE}=6$. *Left panel*: $I_{E}=-0.1$, λ=1. *Right panel*: $I_{E}=-0.5$, λ=1

**Fig. 8:**
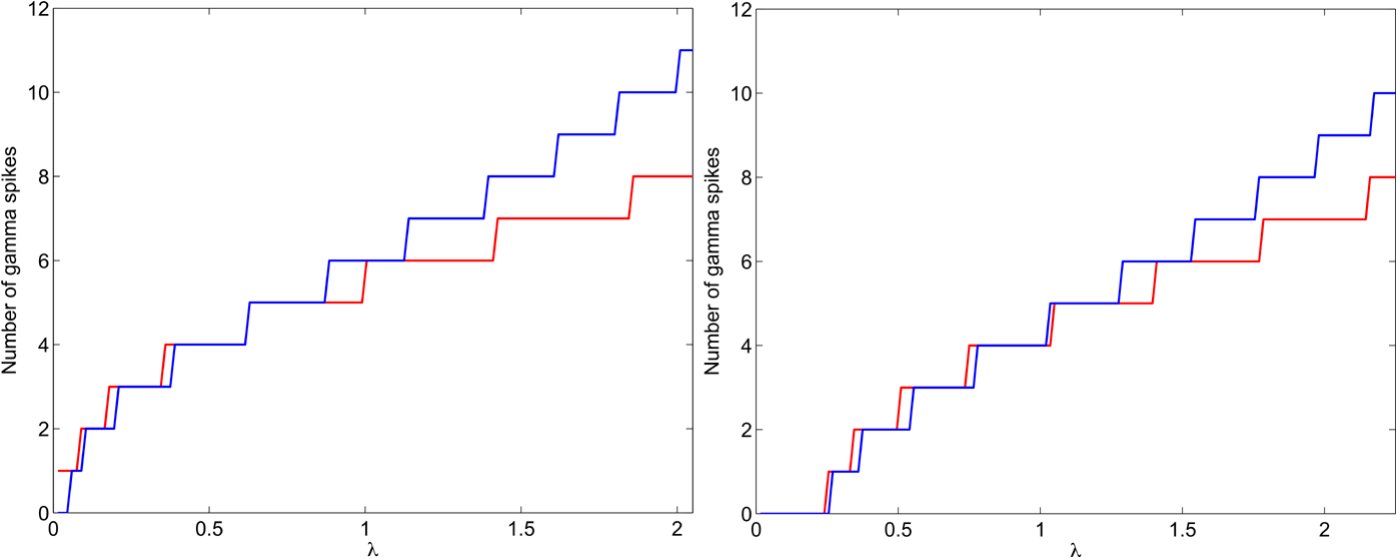
Excitable regime—number of spikes. Number of spikes in the simulation (*blue*) and the prediction of the formula as a function of *λ*. *Left panel*: $I_{E}=-0.1$, $g_{IE}=6$, ε=0.1, ω=4. *Right panel*: $I_{E}=-0.5$, $g_{IE}=6$, ε=0.1, ω=4

This formula works adequately for large inhibition and relatively small (negative) $I_{E}$. Otherwise, due to the intricate interplay between the growth of *Θ* and the decay of *s* almost to 0 (witnessed in the computation of $T_{2}$), it is not sufficient to have just the lowest terms of the *ε* expansion of $\Delta T_{j}$. Figure 7 shows two cases where the formula gives the exact prediction of the number of gamma spikes. In Fig. 8, we show the direct comparison between the predictions of the formula and the simulation, as a function of *λ*. For *λ* large inhibition is too weak to time the spikes and the estimate of the formula becomes too small. 

## 7 Conclusions and Future Directions

In this paper, we investigated how a continuous, strong, low frequency (1–10 Hz) modulation determines the spiking properties of a simplified PING oscillator. This work has been particularly motivated by recent investigation on the role of theta-gamma interactions in processing speech signals [52]. Syllabic input are in fact known to possess a quasiperiodic structure matching theta frequency [24]. Within this framework, theta-modulated gamma spikes need to be aligned to the onset and the offset of linguistically relevant chunks [23]. It is then crucial to understand the timing of gamma spikes and the way they are influenced by theta input, since theta is supposed to detect the presence of long timescale syllabic content. It remains to be unveiled whether the scaling we analytically determined here is produced in more realistic models for speech processing [52] currently under development. Indeed, this result could also be used for other purposes: investigating how theta fluctuations modulate gamma firing in the hippocampus; determining the impact of alpha oscillations on higher frequencies (including gamma), which are thought to carry bottom-up information in visual perception. Indeed timing of first spike is assumed to be particularly relevant in visual cortex, since it is has been shown that it would facilitate the neural encoding of stimuli [53]. 

To explore the dynamics of the system, we split the problem into two parameter regimes: In the first, the frequency of gamma spikes is only modulated by theta phase, while in the second the gamma cell would only fire if forced by theta input. In the former regime, by restating the problem in form of a Mathieu differential equation and looking at the first zero of the Mathieu function solving the initial value problem, we were able to find the time to first gamma spike. In the latter, we separate the dynamics into three time scales, one characterizing EG neuron dynamics in absence of any external input, one for theta dynamics, and one for synaptic inhibition, and we approximate the time to first spike by using an extension of the geometric singular perturbation theory based on the application of the blow-up method [46,48]. 

Computations align with the intuition (arising from the fact that $\theta_{E}$ is a type I neuron) that time to first spike decreases in both cases with coupling strength *λ* and constant driving current $I_{E}$. Interestingly, in the excitable case, we found that time to first spike depends approximately on $\lambda^{-1/6}$, which implies that it saturates rapidly as *λ* grows. As a second notable result, $T_{1}$ scales as $c_{0}/\varepsilon_{\Theta }+c_{1}/\varepsilon_{\Theta }^{1/3}+O(\ln(\varepsilon_{\Theta }))$, $\varepsilon_{\Theta }$ being the speed of theta cycle. Building on these results, we subsequently computed the time to successive spikes in both regimes, where inhibitory synaptic decay time becomes an important factor. For both regimes were able to compute approximate spike times and predict the exact number of spikes per theta cycle (and instantaneous frequency of firing as a direct consequence) in a range of parameter values that leads to firing within the gamma frequency band.

In the present work, we analyzed a simple system in which coupling was limited to a feedforward theta-gamma connection. It would be a natural next step to extend the analysis to bidirectional coupling by including a feedback from gamma spikes to the *Θ* oscillator. A second assumption we made in constructing our system stated that the gamma circuit internal delay between excitatory and inhibitory spikes was negligible, meaning that both cells would fire at exactly the same time. To make the model more biologically appealing, one could relax this hypothesis by introducing a synaptic delay after an excitatory spike and study the correspondent system (i.e., a full PING). For relatively short delays, we would expect the results obtained in this paper to hold at least qualitatively. Throughout this paper, we considered gamma to be a simplified PING generator, on the other hand it still remains an open question whether the same characteristics of theta-gamma modulation we explored here would still be found in a different gamma generator, e.g., an Interneuron Network Gamma (ING) network [54] that can still be implement with Type I neurons as in the case of this work. 

## Appendix

We show that the term of order $O(\ln\varepsilon_{\Theta })$ in expansion (17) is zero in our model. The subsequent term, of order O(1) is also zero, but we do not include the result here since computations are long and heavy. The interested reader could derive this result from [51]. 

In the absence of any synaptic input from the inhibitory neuron, we restate system (1) to reduce the complexity of notation:

(40) $$\begin{array}{rl} \frac{dx}{dt} & =\varphi (x,y)\equiv \left(1-\cos(x)\right)+\left(I_{E}+\lambda \left(1+\cos(y)\right)\right)\left(1+\cos(y)\right),\\ \frac{dy}{dt} & =\varepsilon \psi (x,y)\equiv \varepsilon \omega . \end{array}$$

Then, following [51], we write the expansion

(41) $$T_{1}=\frac{\Theta_{0}+\pi }{\varepsilon_{\Theta }\omega }+\frac{C_{0}}{\varepsilon_{\Theta }^{1/3}}+D_{0}\ln\varepsilon_{\Theta }+E_{0}+O\left(\varepsilon_{\Theta }^{1/3}\right),$$

which approximates the time to the first spike in the excitable case.

Coefficient $D_{0}$ in (41) might be written as

(42) $$\begin{array}{rl} D_{0}= & \frac{1}{3}\frac{1}{\psi (S)}\frac{6\varphi_{xx}(S)\psi_{x}(S)-2\varphi_{xxx}(S)\psi (S)}{3\varphi_{xx}^{2}(S)}\\ & -\frac{1}{6}\left|\psi (S)\right|\frac{\psi_{x}(S)\sqrt{2/|\varphi_{xx}(S)\varphi_{y}(S)|}}{\psi^{2}(S)\sqrt{\left|\varphi_{xx}(S)/(2\varphi_{y}(S))\right|}}\mathrm{sign}\varphi_{y}(S), \end{array}$$

where *S* stands for the coordinates of the singular point $S=(0,\Theta_{0})$ and subscripts indicate the derivatives, i.e., $\varphi_{x}(S)$ is the first derivative of *φ* with respect to *x*, taken at $\left(0,\Theta_{0}\right)$. It is easy to verify that any derivative of *φ* with respect to *x* of order *n*, for *n* odd, is equal to zero at *S*. Furthermore, since ψ(x,y) is constant in system (40), $\psi_{x}(S)$ is clearly zero. Hence, $D_{0}=0$.

## Competing Interests

The authors declare that they have no competing interests.

## Authors’ Contributions

LF, MK, AH, BG designed research; LF, MK performed research; LF, MK, AH, BG wrote the manuscript.
